# Targeted genome editing restores T cell differentiation in a humanized X-SCID pluripotent stem cell disease model

**DOI:** 10.1038/s41598-017-12750-4

**Published:** 2017-09-29

**Authors:** Jamal Alzubi, Celeste Pallant, Claudio Mussolino, Steven J. Howe, Adrian J. Thrasher, Toni Cathomen

**Affiliations:** 1 Institute for Transfusion Medicine and Gene Therapy, Medical Center – University of Freiburg, Freiburg, Germany; 2 Center for Chronic Immunodeficiency, Medical Center – University of Freiburg, Freiburg, Germany; 3 Institute of Child Health, University College London, London, United Kingdom; 4 Great Ormond Street Hospital, NHS Foundation Trust, London, United Kingdom; 5 Faculty of Medicine, University of Freiburg, Freiburg, Germany; 6 Present Address: GlaxoSmithKline plc., Stevenage, Hertfordshire, United Kingdom

## Abstract

The generation of T cells from pluripotent stem cells (PSCs) is attractive for investigating T cell development and validating genome editing strategies *in vitro*. X-linked severe combined immunodeficiency (X-SCID) is an immune disorder caused by mutations in the *IL2RG* gene and characterised by the absence of T and NK cells in patients. *IL2RG* encodes the common gamma chain, which is part of several interleukin receptors, including IL-2 and IL-7 receptors. To model X-SCID *in vitro*, we generated a mouse embryonic stem cell (ESC) line in which a disease-causing human *IL2RG* gene variant replaces the endogenous *Il2rg* locus. We developed a stage-specific T cell differentiation protocol to validate genetic correction of the common G691A mutation with transcription activator-like effector nucleases. While all ESC clones could be differentiated to hematopoietic precursor cells, stage-specific analysis of T cell maturation confirmed early arrest of T cell differentiation at the T cell progenitor stage in X-SCID cells. In contrast, genetically corrected ESCs differentiated to CD4 + or CD8 + single-positive T cells, confirming correction of the cellular X-SCID phenotype. This study emphasises the value of PSCs for disease modelling and underlines the significance of *in vitro* models as tools to validate genome editing strategies before clinical application.

## Introduction

Pluripotent stem cells (PSCs), such as embryonic stem cells (ESCs) and induced PSCs (iPSCs), are attractive cells for the development of novel, patient-specific approaches in regenerative medicine, drug discovery and disease modelling. While ESCs are derived from the inner cell mass of mammalian blastocysts^1^, iPSCs are generated *in vitro* by the expression of defined transcription factors needed to convert a differentiated somatic cell into pluripotency^2^. Both cell types share common characteristics, such as their ability to grow indefinitely while maintaining pluripotency, and the ability to differentiate into somatic cell types, including blood and immune cells.

T cells are a key component of the adaptive immunity, which provides host protection against pathogens and cancer. Unlike other haematopoietic lineages, T cell development occurs outside the bone marrow in the thymus, a lymphoid organ that provides the optimal microenvironment to support T cell maturation^3^. Patients with hereditary defects in the T cell compartment can be severely immune deficient, and the underlying disorders are collectively called severe combined immunodeficiency (SCID)^4^. One of the most common forms is X-linked SCID (X-SCID), which is caused by mutations in the *IL2RG* gene^5,6^. *IL2RG* codes for the common gamma chain (GC), which is present in several interleukin receptors, such as the IL-2, IL-4, IL-7, IL-9, IL-15 and IL-21 receptors, and therefore essential for the development and function of lymphocytes^7^. The immune phenotype of X-SCID patients is characterized by the absence of T and NK cells in combination with poorly active B cells in their peripheral blood^8^. Because the early block in lymphopoiesis limits readily accessible patient material, X-SCID is difficult to study in patients. Moreover, the available mouse models fail to accurately recapitulate the human phenotype^9^. Thus, a stage-specific *in vitro* generation of T cells from PSCs is a valuable tool to better characterise the cellular phenotype of X-SCID.

X-SCID disease is of particular importance for the assessment of novel genome editing applications as gene therapy approaches for this disorder have been successfully validated in the clinic^10,11^. Retroviral *IL2RG* gene transfer in haematopoietic stem cells (HSCs) has been assessed in autologous settings in several clinical trials. The outcome of these studies has shown near complete immune reconstitution, with similar or even better outcome to that of mismatched allogeneic HSC transplantation^12^. While insertional mutagenesis led to the development of leukaemia in two early gene therapy trials involving first-generation gamma-retroviral vectors^13,14^, more recent trials with self-inactivating (SIN) vectors were successful without severe adverse events so far^10^. Additionally, a pre-clinical proof-of-concept study for zinc-finger nuclease (ZFN)-mediated correction of the *IL2RG* gene in HSCs demonstrated the feasibility of targeted gene editing in such multipotent cells^15^.

Designer nucleases are custom-made genome modifiers that have developed into indispensable tools for modelling human disease *in vitro* and for clinical applications^16^. The major classes of designer nucleases comprise ZFNs^17^, transcription activator-like effector nucleases (TALENs)^18,19^, and the clustered regularly interspaced short palindromic repeats (CRISPR)-Cas system^20^. These nucleases induce a site-specific DNA double strand break that activates one of the two major DNA repair pathways, non-homologous end joining (NHEJ) or homology-directed repair (HDR), which in turn can be harnessed either for gene disruption or gene targeting in the presence of a suitable donor DNA template^21^.

Although HSCs are the most relevant cell type for gene editing geared towards clinical translation, several restraints limit their use for detailed biological analyses, including the lack of robust protocols to culture and expand HSCs *in vitro*. PSCs, on the other hand, provide an unlimited source of stem cells that can be cloned and terminally differentiated into somatic cells of interest^22^. In several studies PSCs were combined with designer nuclease technology to provide proof-of-concept that genome engineering can be used to tackle disorders, including many haematopoietic disorders, such as chronic granulomatous disease^23,24^, β-thalassemia^25^ and haemophilia^26^. With regard to the *in vitro* generation of immune cells, PSCs have been successfully differentiated to myeloid cells^23,24,27,28^, but the *in vitro* production of lymphocytes has proven to be difficult. The differentiation of defined murine or human HSCs to T cells has been successfully achieved by cultivating the stem cells on a monolayer of murine OP9 bone marrow stroma cells expressing the notch delta-like 1 ligand (OP9-DL1)^29^. The same general setup was adapted to differentiate PSCs to T cell precursors, with the generation of few mature T cells alongside immature CD4 + /CD8 + double-positive (DP) T cells^30–33^. Recently, patient-specific *IL2RG*, *JAK3* and *WAS*-deficient iPSC lines were corrected by designer nucleases and combined successfully with NK and T cell differentiation protocols^34–36^. However, these studies did not focus on stage-specific T cell development, which is particularly important when modelling X-SCID in order to demonstrate the role of GC in IL-7 and IL-2 dependent T cell development and T cell expansion^7,37^.

Here, we report a differentiation protocol, which enabled us to generate single-positive T cells from murine ESCs in a stage-specific manner. We verified the potential of this protocol for disease modelling by utilizing ESCs in which the murine *Il2rg* locus was replaced with a human *IL2RG* version harbouring the common G691A mutation in exon 5. After correction of the underlying mutation in *IL2RG* with the TALEN technology, the resulting ESCs were differentiated in the presence of IL-7 and IL-2 to single-positive CD4+ and CD8+ T cells, confirming correction of the cellular X-SCID phenotype *in vitro*. This study re-emphasises the significance of designer nucleases as a tool for targeted genetic engineering and demonstrates that pluripotent stem cells can be used to analyse in-depth the stage-specific differentiation of hematopoietic precursor cells into single-positive T cells.

## Results

### Generation of a humanised X-SCID mouse ESC model

To generate the humanised X-SCID mouse ESC line, the full-length mouse *Il2rg* locus with its promoter and enhancer elements was replaced by a full-length human *IL2RG* locus containing the common G691A point mutation in exon 5. This humanised cellular mouse model of X-SCID not only provides the mutant *IL2RG* target for site-specific endonucleases, but due to its location in the mouse genome, it also takes into account the chromatin structure and epigenetic factors that may affect gene targeting.

### Genetic correction of *IL2RG* locus

**Figure 1:**
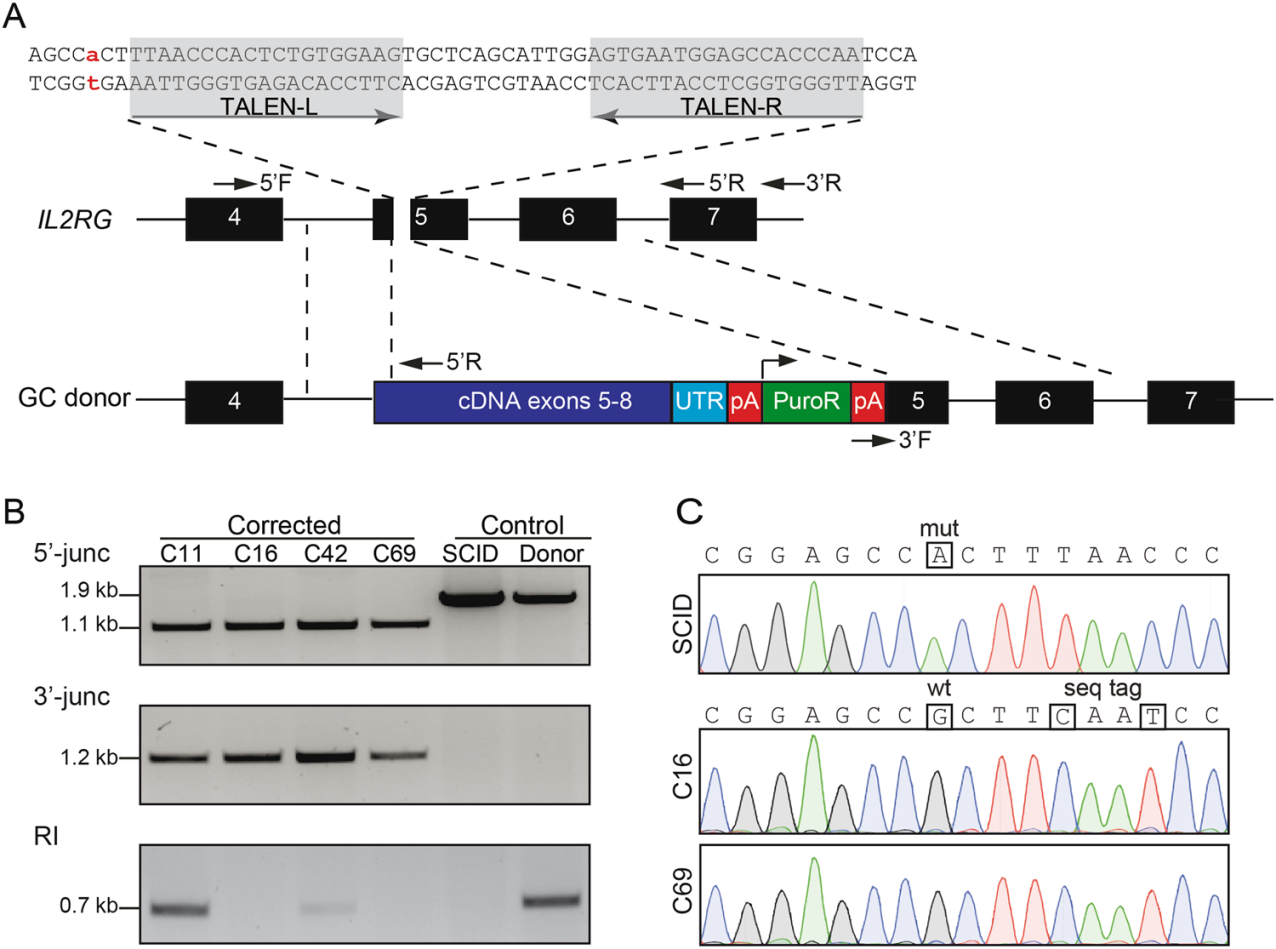
Genetic correction of X-SCID. (**A**) Schematic of targeting strategy to correct the G691A mutation in *IL2RG* exon 5. The point mutation is highlighted in red and the TALEN target site in grey. The GC donor contains the therapeutic super-exon encompassing exons 5 to 8, a puromycin selection (PuroR) cassette, and homology arms to the endogenous *IL2RG* locus (indicated by interrupted lines). Arrows mark the binding sites of the primers used for PCR-based genotyping. (**B**) Genotyping. PCR-based genotyping was performed to detect the 5′-junction (primers 5′F/5′R), 3′-junction (primers 3′F/3′R; see also Figure S1) and randomly integrated (RI) donors (primers RIF/RIR; see also Table S1). Uncropped gel images shown in Figure S4B. (**C**) Sequence analysis of exon 5. The position of the mutated ‘A’ and the reversion to ‘G’ as well as the sequence tag (seq tag) are highlighted. C11, C16, C42, C69, corrected ESC clones; SCID, uncorrected X-SCID ESC clone; Donor, ESC clone transfected with donor alone.

In previous studies, we compared the activities and toxicities of ZFNs and TALENs targeting exon 5 of the human *IL2RG* locus^38,39^. One specific TALEN pair combined high activity with little cytotoxic and was therefore chosen for this study. To correct the mutation in exon 5, we generated a therapeutic donor construct (GC donor) that contained a partial cDNA coding for exons 5 to 8, followed by the natural 3′-untranslated region (UTR) and a polyadenylation (pA) site (Fig. 1A). The inclusion of the natural 3′-UTR in combination with a viral pA site should stabilise the mRNA and improve transcriptional termination in order to enhance expression of the *IL2RG* gene product. To prevent cleavage of the donor by the TALEN pair, two silent mutations (sequence tag) were introduced into the left TALEN binding half-site. Furthermore, the donor harboured a phosphoglycerate kinase (PGK) promoter driven puromycin resistance cassette (PuroR) to enable enrichment of gene targeted cells. X-SCID ESCs were transfected with TALEN expression plasmids and the GC donor. Following puromycin selection, 100 resistant ESC clones were expanded and screened for the targeted integration event. Initial screening involved PCR-based confirmation of the 3′-junction and the presence of the donor (Figs 1B,S1). The expected PCR amplicon was detected in four out of 100 clones, indicative of successful integration of the GC donor into the endogenous *IL2RG* locus (Figure S1). Further genotyping involved a PCR-based confirmation of the correct 5′-junction in these four clones (Fig. 1B). The expected PCR amplicon of 1.1 kb was detected in all four clones, whereas the 1.9 kb product, indicative of the ‘SCID allele’, was detected only in non-transfected samples (mock) and cells transfected with the GC donor alone (Fig. 1B). To identify non-targeted random integration (RI) events, a PCR with a primer binding in the backbone of the GC donor was applied. Two of the four clones were negative, showing that these corrected clones (C16, C69) do not harbour additional integration events (Fig. 1B). For further proof of genetic correction, exon 5 was sequenced. Sequence analysis of both corrected clones confirmed correction of the point mutation and the presence of the sequence tag (Fig. 1C). In conclusion, we were able to genetically correct a point mutation in the human *IL2RG* gene in pluripotent stem cells using TALEN-mediated targeted integration of a partial cDNA into exon 5 of this locus.

### Differentiation of ESCs to CD4 + /CD8 + double-positive T cells

In order to induce T cell development from haematopoietic stem cells (HSCs) *in vitro*, a combination of notch and IL-7 mediated signalling is essential^29,40,41^. Initial experiments with bone marrow derived lineage-negative (lin-) cells showed that co-cultivation with OP9-DL1 stroma cells enabled the differentiation of these multipotent stem cells through all CD4-/CD8- double-negative (DN) stages, as determined by flow cytometric analysis of CD25 and CD44 surface expression (Figure S2). Further cultivation of these early thymocytes on OP9-DL1 cells led to the generation of CD4+/CD8+ double-positive (DP) T cells, which expressed the beta-chain of the T cell receptor (TCRβ), indicating that these cells have successfully passed the β-selection checkpoint *in vitro*.

**Figure 2:**
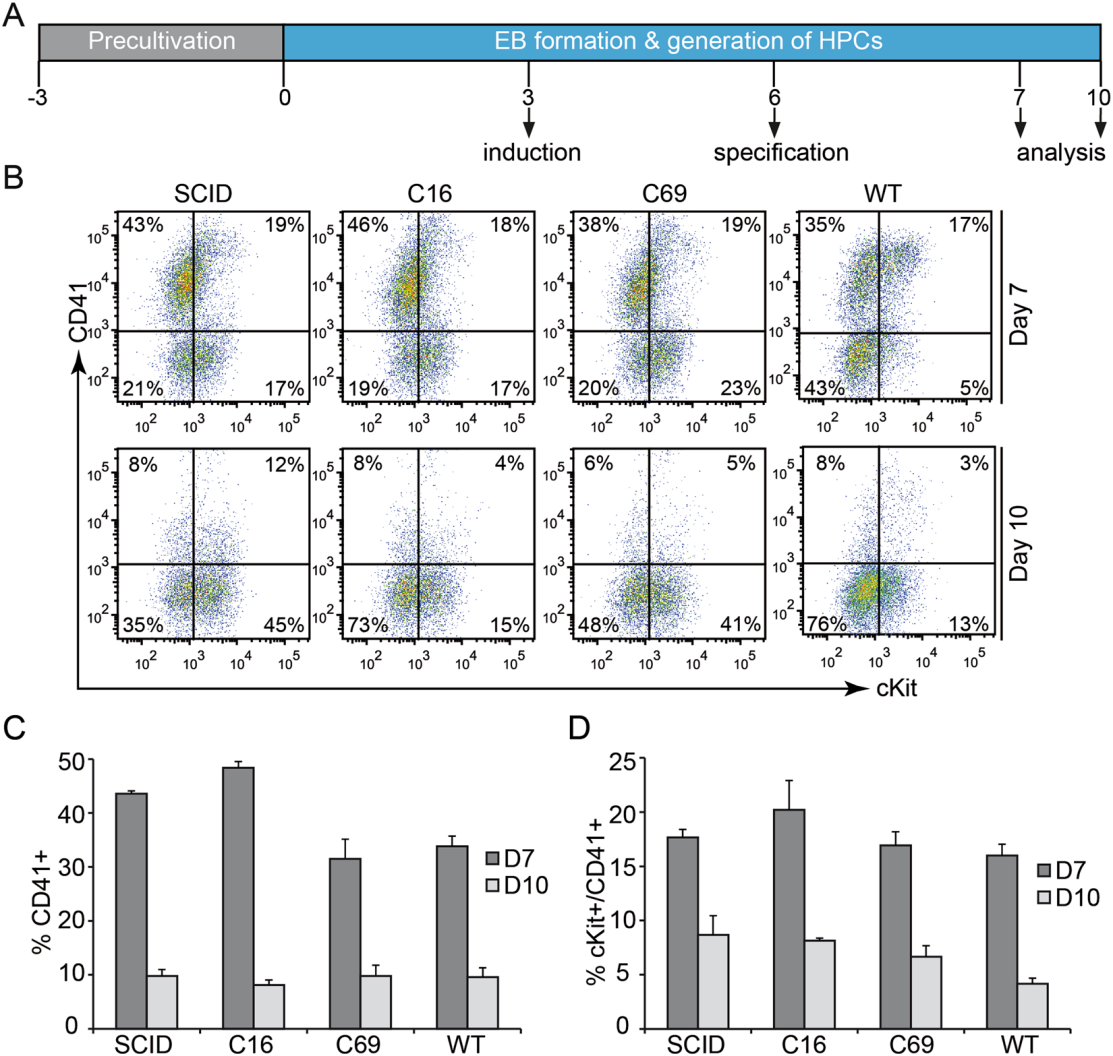
Differentiation of ESCs to hematopoietic precursor cells. (**A**) Schematic overview of the differentiation protocol. Humanised mouse ESCs were kept in pluripotent state in mESC medium (precultivation). For EB formation and generation of hematopoietic precursor cells (HPCs), ESCs were cultured for either 7 or 10 days in EB medium. EBs were primed with mesoderm-inducing cytokines at day 3 (induction) and with haematopoiesis-inducing cytokines at day 6 (specification). (**B**) Qualitative assessment of *in vitro* generated HPCs. EBs were dissociated and stained for CD41 and c-Kit (CD117) at days 7 and 10. Flow cytometric analysis is shown for 7-AAD negative cells. Numbers indicate percentage of cells in each quadrant. (**C,D**) Quantitative assessment of *in vitro* generated HPCs. Shown are the average percentages with standard error of mean (S.E.M.) of generated CD41+ (**C**) or cKit+/CD41+ (**D**) cells harvested at days 7 or 10 (n = 3). SCID, uncorrected X-SCID ESC clone; C16 and C69, corrected ESC clones; WT, wildtype mouse ESC clone.

Pluripotent stem cells have the potential to differentiate to defined somatic cell types only when the right differentiation conditions are available at the right time. Differentiation of pluripotent stem cells to T cells includes an initial mesoderm induction, followed by the specification to haematopoietic precursor cells (HPCs) before proceeding to T cell differentiation (Fig. 2A). To this end, we have established an embryoid body (EB)-based differentiation protocol to generate HPCs from corrected and non-corrected X-SCID ESC clones, as well as from the wildtype (WT) ESC clone CCE^1^ as a control. In order to monitor the optimal time point for HPC induction, EBs were harvested and dissociated either directly after hematopoietic specification at day 7, or three days later at day 10. Enrichment of HPCs was monitored by assessing the expression of the surface markers CD41 and c-Kit (CD117)^42^ (Fig. 2B). For all ESC lines, comparable levels of CD41+or cKit+/CD41+ cells were detected at day 7 (Fig. 2C,D). However, the fraction of CD41+ or cKit+/CD41+ cells dropped sharply when harvested at day 10, and we were not able to differentiate these ‘late’ HPCs into T cells (not shown). This indicates the importance of proceeding to T cell differentiation directly after specification to hematopoietic differentiation at day 7, where a prominent cKit+/CD41+ cell population is evident.

**Figure 3:**
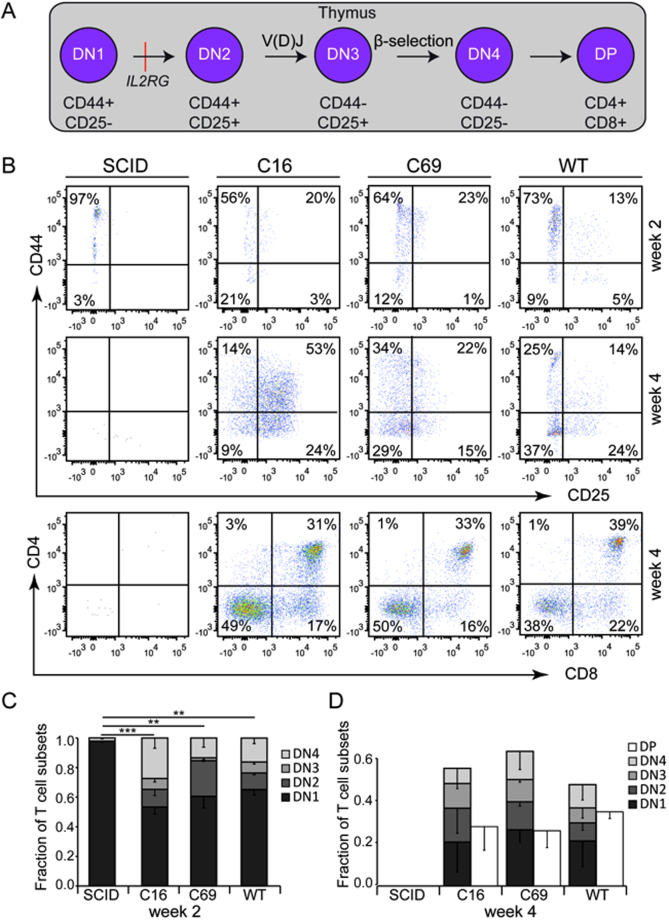
*In vitro* differentiation of ESC-derived hematopoietic precursors to CD4+/CD8+ double-positive T cells. (**A**) Schematic of early stage T cell development. ESCs derived hematopoietic precursor cells (HPCs; Fig. 2) were cultured on OP9-DL1 cells to provide DL-1 mediated notch signalling that is needed to commit differentiation of HPCs to the T cell lineage, including CD4-/CD8- double-negative (DN) early T cell progenitors (DN1), pro-T cells (DN2), which undergo V(D)J recombination to become pre-T cells (DN3), that in turn pass β-selection to develop into DN4 and finally CD4+/CD8+ double-positive (DP) T cells, which express the pan-lymphocyte marker CD3. Absence of the *IL2RG* gene product leads to an early block in T cell development at DN1 stage (red line). (**B**) Qualitative assessment of T cell maturation. Differentiation was analysed by flow cytometry after two and four weeks of co-cultivation on OP9-DL1 stroma cells by monitoring surface expression of CD44/CD25 and CD4/CD8 (see also Figure S2). (**C,D**) Quantitative assessment of T cell maturation. Shown is the average fraction of the various T cell subpopulations with standard error of mean (S.E.M.) of three independent experiments of cells analysed at week 2 (**C**) or week 4 (**D**). DN T cell subsets add up to 1.0 at week 2 (**C**), while at week 4 (**D**) also CD4 + /CD8 + DP (white columns) and some single-positive cells (not indicated, see Fig. 4) were present. Statistical differences in the fraction of DN1 cells at week 2 are indicated by **(p < 0.01) and ***(p < 0.001). No statistical differences between any of the generated T cell subsets derived from the gene-edited ESC lines and the WT clone was observed at week 4 (p > 0.05). Gating was applied in the following order: FSC/SSC and CD45 + /DAPI- (not shown) to assess CD4/CD8 expression; CD4-/CD8- to gate for DN1-DN4 stages assessed by CD25/CD44 expression. SCID, uncorrected X-SCID ESC clone; C16 and C69, corrected ESC clones; WT, wildtype ESC clone.

Because the *IL2RG* gene product is an integral part of the IL-7 receptor, it is essential for early T cell development. We therefore hypothesised that HPCs derived either from corrected ESC clones or WT ESCs will be able to differentiate to DP T cells, while cells derived from the non-corrected X-SCID ESC clone would be blocked at the DN1 stage (Fig. 3A). To verify this notion, day 7 HPCs were co-cultivated on a monolayer of OP9-DL1 cells, and T cell maturation profiled on a weekly basis by flow cytometry. We monitored the different developmental stages by determining surface expression of CD44 and CD25 within the CD4-/CD8- DN population: DN1 (CD44+/CD25-), DN2 (CD44+/CD25+), DN3 (CD44-/CD25+) and DN4 (CD44-/CD25-). As hypothesised, maturation of thymocytes derived from non-corrected HPCs was blocked at the DN1 stage (97%) and only detectable at week 2 of the analysis but not week 4 (Fig. 3B–D), suggesting that the derived T cell precursors go into apoptosis if differentiation is blocked at an early stage. Conversely, thymocytes derived from both corrected ESC clones (C16, C69) and the WT clone could progress into DN2, DN3, and DN4 stages **(**Fig. 3B,C **)**. Moreover, after 4 weeks of cultivation, the development of CD4+/CD8+ DP T cells became evident **(**Fig. 3B,D **)**, similarly to the T cells derived from lin- cells (Figure S2). Statistical analyses confirmed a difference in the fraction of generated DN1 T cells derived from the SCID clone as compared to all other clones at week 2 **(**Fig. 3C **)** but not any significant differences when comparing any of the generated T cell subsets derived from the genetically corrected ESC lines with the WT clone **(**Fig. 3D **)**. In summary, these results demonstrate that our *in vitro* differentiation protocol allowed us to recapitulate the cellular X-SCID phenotype *in vitro* and enabled us to generate CD4+/CD8+ DP T cells in a stage-specific manner.

### Generation of CD4+ and CD8+ single-positive T cells

**Figure 4:**
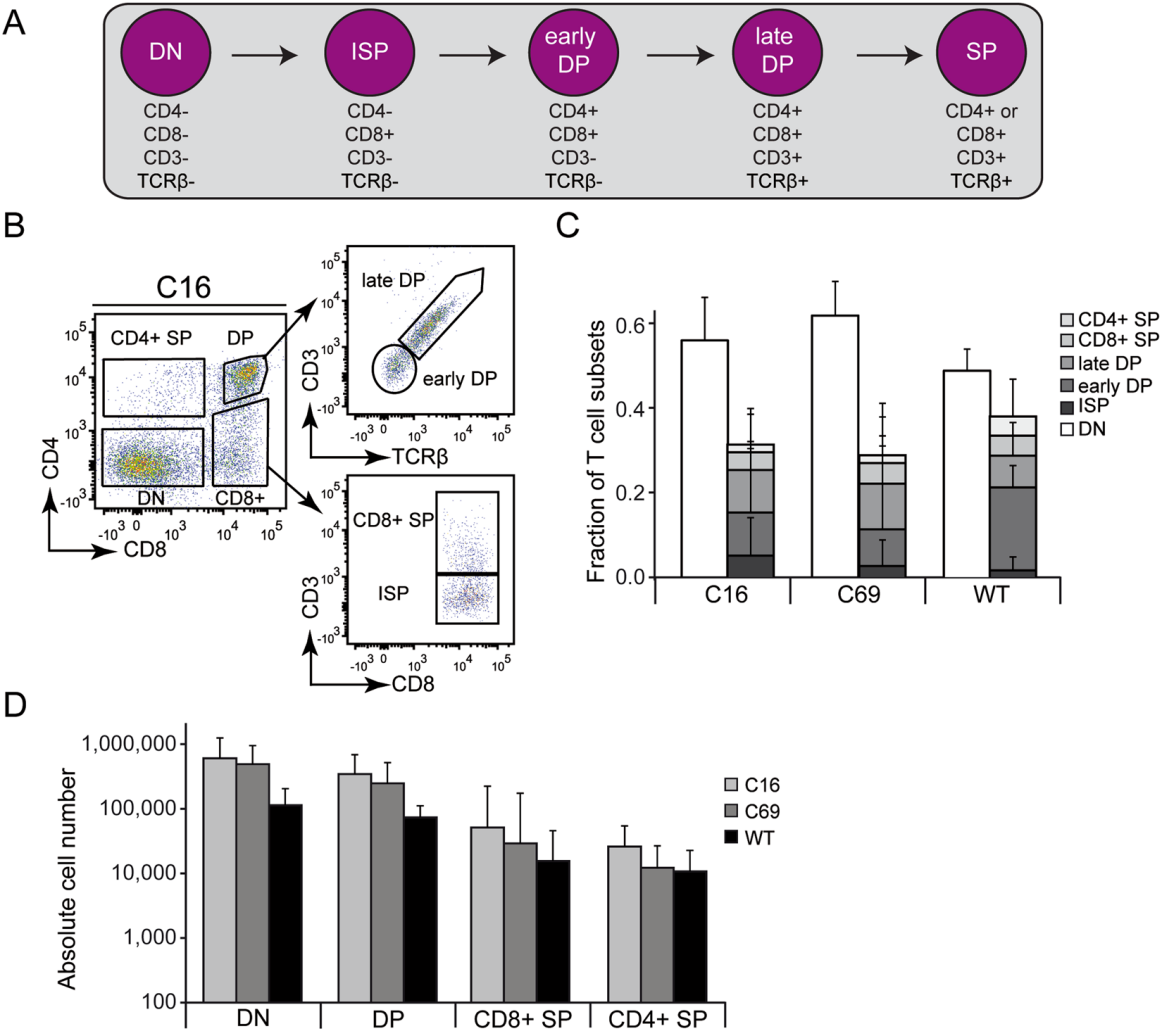
Assessment of *in vitro* generated T cells. (**A**) Schematic of late stage T cell maturation. T lymphocytes generated at week 4 on OP9-DL1 cells were characterised for their maturation stage based on the expression of CD4/CD8 as well as CD3 and T cell receptor beta-chain (TCRβ). CD4-/CD8- double-negative (‘DN’) T cells develop into immature single-positive (ISP) CD8+ cells that then differentiate into ‘early DP’ (CD4+ /CD8+ ) T cells. After passing positive selection, CD3 and TCRβ expression is upregulated. These ‘late DP’ then develop in CD4 + or CD8 + single positive (SP) T cells. (**B**) Qualitative assessment of late T cell maturation. Differentiation was analysed by flow cytometry by monitoring surface expression of CD4/CD8, CD3/TCRß, and CD3/CD8. A representative flow cytometry analysis for ESC clone C16 is shown. Gating was applied in the following order: FSC/SSC and CD45+/DAPI- (not shown) to assess CD4/CD8 expression; CD3 and TCRβ expression assessed on CD8+/CD4+T cells and CD3 expression assessed on CD8+ cells. (**C**) Relative assessment of T cell differentiation. Shown is the average fraction of the various T cell subsets with standard error of mean (S.E.M.) for both gene-edited clones (C16, C69) and the wildtype (WT) ESC clone. No statistical differences between any of the generated T cell subsets derived from the gene-edited ESC lines and the WT clone was observed (p > 0.05). (**D**) Quantitative assessment of the efficiency of T cell differentiation. Shown is the average of three independent experiments with S.E.M. for both corrected clones (C16, C69) and the WT ESC clone. No statistically significant differences between the number of T cell subsets generated from clones C16/C69 and WT was observed (p > 0.05).

We further analysed the week 4 lymphocyte population (Fig. 3) for late T cell maturation stages by assessing the expression of the CD3 complex and the T cell receptor beta chain (TCRβ) (Fig. 4A). In combination with the CD4/CD8 expression patterns, this allowed us to determine the fraction of ‘DN’ T cells, immature single-positive (ISP) cells, ‘early DP’ and ‘late DP’ T cells, as well as CD4+ and CD8+ single positive (SP) T cells (Fig. 4B). When comparing the T cell maturation profiles of the two corrected ESC clones (C16, C69) with the wildtype (WT) cells, we neither observed significant qualitative (Fig. 4C) nor quantitative (Fig. 4D) differences between the generated T cell subsets derived from gene-edited (C16, C69) versus WT ESCs (p > 0.05). The lower numbers of overall produced T cells or the slightly higher percentage of single positive cells produced by the WT clone could be due to genetic differences between the WT cells and the two corrected X-SCID clones, which e.g. contain additional genetic elements in the *IL2RG* locus (Fig. 1A). Nonetheless, these results indicate that the corrected X-SCID cells were able to pass the positive selection checkpoint, which leads to upregulation of the CD3 complex before the cells differentiate into CD4+ and CD8+ single-positive (SP) thymocytes.

To assess whether the genetically corrected T cells are able to respond to IL-2, we activated the week 4 lymphocyte population with anti-CD3/CD28 beads and challenged them with antigen-presenting cells (irradiated splenocytes). After one week of co-culture in the presence or absence of IL-2, we assessed the proliferation capacity of the *in vitro* generated T cell population by labelling the cells with carboxyfluorescein succinimidyl ester (CFSE). Flow cytometric analysis revealed more cell divisions when cells were co-stimulated with IL-2 (Figure S3A). We also evaluated the fraction of CD8+ SP T cells by flow cytometry. While some CD8+ T cells were detectable after stimulation with anti-CD3/CD28 beads alone, IL-2 enhanced the proliferation of SP CD8+ cells considerably (Figure S3B) and induced a strong up-regulation of the IL-2 receptor alpha-chain (CD25) (Figure S3C). In conclusion, these supplementary experiments corroborated that genetic correction of the *IL2RG* locus restored IL-2 dependent proliferation of the T cell population.

## Discussion

The therapeutic potential of haematopoietic stem cells has been extensively validated by allogeneic HSC transplantation for various indications. Moreover, retroviral gene therapy in autologous HSCs has been successfully adapted to treat several primary immunodeficiencies^43^, including X-SCID^10^, adenosine deaminase deficiency (ADA-SCID)^44^, and Wiskott-Aldrich Syndrome (WAS)^45,46^. While clinically relevant, HSCs are only of limited use for *in vitro* disease modelling. In this regard, PSCs have many advantages over HSCs, including the unlimited proliferation potential and the possibility to expand and screen individual clones^47^. Moreover, these cells hold great promise for the development of autologous cell therapies, personalised drug discovery and disease modelling. Here, we developed a cellular X-SCID disease model based on a murine knock-in ESC line that harbours a mutated copy of the human *IL2RG* locus. To model the cellular disease phenotype, we have established a T cell differentiation protocol that enabled us to differentiate early T cell precursors in a stage-specific manner to single positive T cells *in vitro* after TALEN-mediated correction of the *IL2RG* locus.

For clinical translation, it is imperative to identify designer nuclease with high activity, high specificity and low cytotoxicity and genotoxicity. We previously developed a TALEN architecture that combines high activity with high specificity and low cytotoxicity^38^, and this platform was successfully used to generate a TALEN pair to target exon 5 of *IL2RG* ^39^. However, targeting the human *IL2RG* locus with TALENs seems challenging. We have previously reported that the success rate of generating functional TALEN pairs to target *IL2RG* was considerably lower than producing TALENs that cleave the *CCR5* locus or *AAVS1* ^39^. In this study, we used a murine ESC line that contains a mutated human *IL2RG* locus in lieu of the endogenous mouse *Il2rg*. The targeting efficiency after selection was only ~4% (4 out of 100 selected clones). Similarly, a recently reported TALEN-mediated gene targeting frequency in X-SCID patient-derived iPSCs was rather modest^48^. In contrast, ZFN-mediated gene targeting at the *IL2RG* locus in unselected multipotent HSCs reached ~6%^15^. Although the overall low gene targeting frequency in PSCs could be attributed to the potential differences in the chromatin structure at the *IL2RG* locus in PSCs as compared to HSCs, several studies in ESCs and iPSCs confirmed that even non-expressed genes in a closed chromatin structure could be easily targeted by ZFNs and TALENs^49,50^. On the other hand, it was shown that DNA methylation could have a negative impact on target site binding by TALENs^51,52^, which was not observed for ZFNs so far. Further studies will be needed to clarify this point.

Although T cell development is similar between humans and mice^53^, the available mouse disease models for X-SCID have failed to fully recapitulate the human phenotype^54^. This supports the notion that there are other, yet undiscovered molecular mechanisms, which differ between human and mouse. Interestingly, unlike the X-SCID mouse model, we did not observe leakage in T cell maturation in our *in vitro* differentiation system based on the *IL2RG* mutant mouse ESCs. Based on this observation, it will be interesting to generate novel mouse strains from our mutant and corrected ESC lines to address further the differences between *in vivo* and *in vitro* T cell development.

A detailed analysis of the different stages of T cell development *in vitro* is particularly valuable to study T cell maturation in a disease setting or to examine subtle changes in the epigenome or transcriptome during T cell differentiation. To our knowledge, this is the first report that recapitulates T cell differentiation in a disease context through all stages from early thymic progenitors (DN1) to CD4 + and CD8 + single positive T cells. Several studies described the generation of CD4+/CD8 + double positive T cells^55,56^ or even CD4+ and/or CD8+ single positive T cells^30–32^ from either normal murine or mouse ESCs or iPSCs. For X-SCID, Verma and colleagues validated the potential to differentiate gene edited patient derived iPSCs to NK cells and CD4+/CD8+ double positive T cells^48^, but failed to demonstrate stage-specific differentiation and maturation to single-positive T cells. Similarly, two other published studies demonstrated the feasibility to differentiate gene edited *JAK3*-deficient or *WAS* iPSCs to phenotypically mature T cells^35,36^ but did not assess the different stages of T cell development. Here, we show that PSCs can be differentiated in a stage-specific manner from early thymic progenitors (DN1) to single-positive CD4+ or CD8+ T cells. Using ESCs that contain the G691A point mutation in exon 5 of the human *IL2RG* locus, we demonstrate that hematopoietic differentiation of X-SCID ESCs was blocked at the DN1 stage, hence recapitulating the cellular *in vivo* phenotype *in vitro*. Moreover, HPCs derived from gene edited and WT ESCs went through all stages of T cell development, including β-selection, and could be differentiated to single-positive CD4+ or CD8+ T cells. Together, this study indicates that disease modelling based on PSC technology is not only a useful tool to study rare T cell disorders but, in combination with genome engineering tools, will allow researchers to analyse T cell differentiation and maturation step-by-step in an easily trackable *in vitro* system. A restriction in our hands, however, was that T cell development from PSCs seems to be a stochastic event, advocating for the need to identify the correct sub-population within the generated HPCs with high capacity for T cell differentiation.

In conclusion, our study provides evidence that naïve pluripotent stem cells can be differentiated *in vitro* in a stage-specific manner to CD3-positive CD8+ single-positive T cells. In our hands, the established system has been validated successfully for cellular disease models of X-SCID and RS-SCID^33^. Based on the developed protocol, it would be interesting to validate further this protocol to cover a broad range of T cell deficiencies, including mutations in the genes encoding ZAP70, CD3Z and CD45, in order to address different blocks in T cell development. Moreover, in combination with designer nucleases-based gene editing, the technology will also expedite the study of genotype-phenotype correlations and allow for screening of personalised patient-specific drugs by creating disease models in isogenic backgrounds.

## Methods

### Cells and cell culture

All cells were free of mycoplasma and kept at 37 °C with 5% CO2. OP9-DL1 cells (obtained from Juan Carlos Zúñiga-Pflücker) were cultured in OP9-DL1 medium [alpha-MEM (Life Technologies, supplement with 20% FBS (PAA), 100 U/ml of penicillin and 100 µg/ml of streptomycin (Sigma-Aldrich), 2 mM of L-glutamine (Merck Millipore) and 1 mM Na-pyruvate (PAA)]. All ESC lines were cultivated feeder-free on plates coated with 0.1% gelatine in ESGRO complete medium (Merck Millipore), supplement with 100 U/ml of penicillin and 100 µg/ml of streptomycin in addition to 4 µM of SB431542, 1 µM of PD0325901 and 3 µM of CHIR99021 (all Axon Medchem). Lineage-negative HSCs were isolated by flushing tibiae and femurs of C57BL/6 N mice, and purified by magnetic cell sorting (MACS) according to the manufacturer’s instructions (Lineage Cell Depletion Kit, Miltenyi Biotech). All procedures involving the usage of animals have been conducted according to institutional guidelines, national and state regulations, and approved by the Department for Rural Affairs and Consumer Protection of the Federal State of Baden-Wuerttemberg (MLR).

### Plasmids

The TALEN expression plasmids were described before^38,39^. The GC donor plasmid generated by cloning the cDNA PCR product from exon 5–8, followed by the natural 3′-UTR of the *IL2RG* locus (kindly provided by Axel Schambach, Hannover Medical School). To improve transcriptional termination, the HSV polyA was cloned downstream of the 3′-UTR. The homology arms (HAR, HAL) were generated by PCR amplification of the *IL2RG* locus from genomic DNA of normal fibroblasts. Complete sequences and maps of plasmids can be obtained upon request.

### Generation and genotyping of gene targeted mouse ESC clones

The humanised X-SCID mouse ESC line was generated by InGenious as follows: A 5.5 kb region of the human *IL2RG* gene was subcloned from a human BAC clone into shuttle vector pSP72 (Promega). *Mlu*I sites were engineered at the 5′ and 3′ ends by Red/ET recombineering. The G691A mutation was incorporated into exon 5 of the human *IL2RG* locus by overlap-extension PCR. A 14.4 kb region containing the mouse *Il2rg* locus was subcloned from a C57BL/6 BAC clone (RP23: 263O9) into the targeting vector. This region encompassed a 7.1 kb long 5′ homology arm, the 5.1 kb long coding region of the *Il2rg* gene, and the 3′ homology arm of 2.3 kb. The targeting vector also included a neomycin resistance gene for selection. After delivery of this vector to murine ESCs by electroporation, successfully targeted cells were enriched for by neomycin treatment. The aminoglycoside 3′-phosphotransferase cassette was flanked with LoxP sites, allowing the neomycin resistance gene to be excised from the genome via Cre-Lox recombination. Successfully gene targeted ESC clones were sequenced to confirm the presence of the mutant human IL2RG allele and deletion of the neomycin resistance cassette.

One day prior to the experiment, X-SCID ESCs were pre-treated with 10 nmol/ml of ROCK inhibitor (Wako, Japan). 2 × 10e6 cells were nucleofected with 1.25 µg of each TALEN expression plasmids and 2.5 µg of the GC donor using Mouse ES kit and program A-023 (Nucleofector I, Lonza). Selection with 0.4 µg/ml of puromycin (Roth, Germany) was applied for 5 days before individual resistance clones were isolated and expanded in 96-well plates. Once confluent, cells were lysed in 100 µl of Direct Lysis Buffer (PeqLab) supplement with protease K (20 µg/ml) (Thermo Scientific), incubated overnight at 55 °C, followed by heat inactivation at 86 °C for 45 min. 2 µl of the lysis mix was used as a template for the various PCR-based genotyping reactions. All PCR reactions were performed using Phire Hot Start II polymerase (Thermo Scientific, Germany) according to the manufacturer’s guidelines. The used primers are indicated in Supplementary Table 1.

### Generation of CD4+ /CD8+ double-positive T cells

The protocol was modified from our previously published protocol^33^ as follows: For embryoid body (EB) formation, 1 × 10e5 mouse ESCs were kept in EB-medium [IMDM basic medium supplemented with 15% ES cult FBS (StemCell Technologies), 5% PFHMII medium (Life Technologies), 100 U/ml of penicillin and 100 µg/ml of streptomycin (Sigma-Aldrich), 2 mM L-glutamine (Merck Millipore), 50 µg/ml of vitamin C (Sigma-Aldrich), 150 mM monothioglycerol (Sigma-Aldrich), and 200 µg/ml of human transferrin (Sigma-Aldrich)] at 37 °C in 5% CO2 on a platform shaker at 80 rpm for a total of 7 to 10 days. Mesoderm induction was initiated at day 3 with rhBMP4, activin A, rhFGF-2 and rhVEGF (all at a final concentration of 5 ng/ml; all R&D Systems), followed by hematopoietic specification at day 6 with mIL-3 (10 ng/ml) and mSCF (40 ng/ml) (both Peprotech). To harvest the EBs, cells were incubated in 2 ml TrypLE (10X, Life Technologies) for 20 min at 37 °C incubator before being passed through 70 µm mesh. To induce T cell differentiation, the single cell solution containing HPCs was cultivated on a fresh monolayer of OP9-DL1 cells and primed with 2 ml of T cell medium [OP9DL1 complete medium supplemented with 1 ng/ml of mIL-7 and 5 ng/ml of rhFlt3-L (both Peprotech)]. Cells were harvested weekly by passing through a 70 µm mesh before being transferred to fresh monolayer of OP9-DL1 cells. The extent of T cell differentiation was assayed by flow cytometry as described below.

### Generation of CD8+ single-positive T cells and cell proliferation assay

Mice splenocytes derived from C57BL/6 N mice were isolated and irradiated at 30 Gy prior to use. CD4+/CD8+ DP T cells were stained with 0.25 µM of carboxyfluorescein succinimidyl ester (CFSE) according to manufacturer’s instructions (Life Technologies). 5 × 10e5 stained cells were co-cultivated with the irradiated splenocytes at a ratio of 1:1 in 2 ml of T cell medium supplemented with anti-CD3/CD28 beads (Miltenyi Biotech) at a cell-to-bead ratio of 1:2 in the presence or absence of mIL-2 (100 ng/ml final volume). After 7 days, cells were harvested and analysed by flow cytometry as described below.

### Flow cytometry

For flow cytometric analysis, cells were resuspended in FACS buffer (PBS supplemented with 2% FCS, 1 mM EDTA and 0.1% sodium azide (both Sigma-Aldrich)). EB-derived HPCs were treated with mouse Fc-blocking antibodies (BD Biosciences, Cat# 553142) for 5 minutes at 4 °C prior to staining with anti-CD41-PE (Cat# 12-0411-81) and anti-cKit-APC (Cat# 17-1171-81) antibodies, or corresponding isotype controls respectively (all eBioscience), and stained with 7-AAD for 2 min before analysis on BD Accuri C6 flow cytometer (BD Biosciences). ESC-derived T cells were stained with anti-CD45-APC-Cy7 (Cat# 557659), anti-CD4-PerCPR-Cy5.5 (Cat# 550954), anti-CD8-PE-Cy7 (Cat# 552877) (all BD Biosciences), anti-CD44-PE (Cat# 12-0441-81), anti-CD25-APC (Cat# 17-0251-81), anti-TCRß-FITC (Cat# 11-5961-82), anti-CD3e-PE (12-00331-82) (all eBioscience) antibodies and DAPI, before analysis on a FACSCanto II (BD Biosciences). All samples were evaluated using FlowJo software (Tree Star). To eliminate OP9-DL1 stroma cells from analysis, all samples were gated for CD45+ cells.

### Statistical Analysis

All experiments were performed at least three times. Error bars represent standard error of mean (S.E.M.). Statistical significance was determined with a two-tailed Student’s *t*-test with equal variance.

### Data Availability Statement

All data generated or analysed during this study are included in this published article (and its Supplementary Information files).

## Electronic supplementary material

Supplementary Information
