# Supplementary material for: Targeted genome editing restores T cell differentiation in a humanized X-SCID pluripotent stem cell disease model
Source: Sci Rep. 2017 Sep 29;7:12475. doi: 10.1038/s41598-017-12750-4 (PMC5622068; doi:10.1038/s41598-017-12750-4)
Supplement: Supplementary file 1 — Supplementary Information [file 41598_2017_12750_MOESM1_ESM.pdf]

SUPPLEMENTARY INFORMATION

Targeted genome editing restores T cell differentiation in a humanized X-SCID pluripotent stem cell disease model

Jamal Alzubi[1,2], Celeste Pallant[3,#], Claudio Mussolino[1,2], Steven J. Howe[3,#], Adrian J. Thrasher[3,4] and Toni Cathomen[1,2,5,*]

[1] Institute for Transfusion Medicine and Gene Therapy, Medical Center – University of Freiburg, Freiburg, Germany

[2] Center for Chronic Immunodeficiency, Medical Center – University of Freiburg, Freiburg, Germany

[3] Institute of Child Health, University College London, London, United Kingdom

[4] Great Ormond Street Hospital, NHS Foundation Trust, London, United Kingdom

[5] Faculty of Medicine, University of Freiburg, Freiburg, Germany

[#] Current address: GlaxoSmithKline plc., Stevenage, Hertfordshire, United Kingdom

Item List

Figure S1. Screening for corrected X-SCID ESC clones.
Figure S2. *In vitro* T cell development of lineage-negative cells.
Figure S3. Response of *in vitro* generated T cells to IL-2.
Figure S4. Uncropped images.
Table S1. Primer used for genotyping

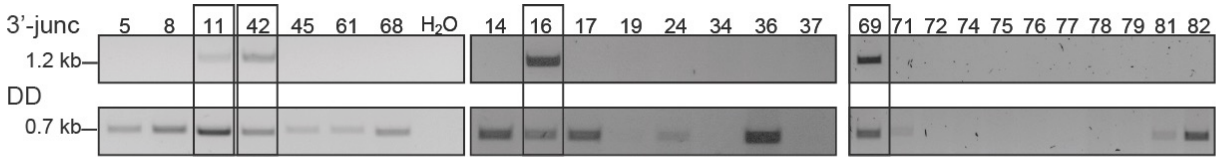

Figure S1. Screening for corrected X-SCID ESC clones. PCR-based genotyping was performed to detect 3'-junction (primer pair 3'F/3'R) and donor integration (primer pair DDF/DDR; see Table S1). Numbers on top indicate individual clone numbers. Positive clones are boxed. Uncropped images shown in Figure S4A.

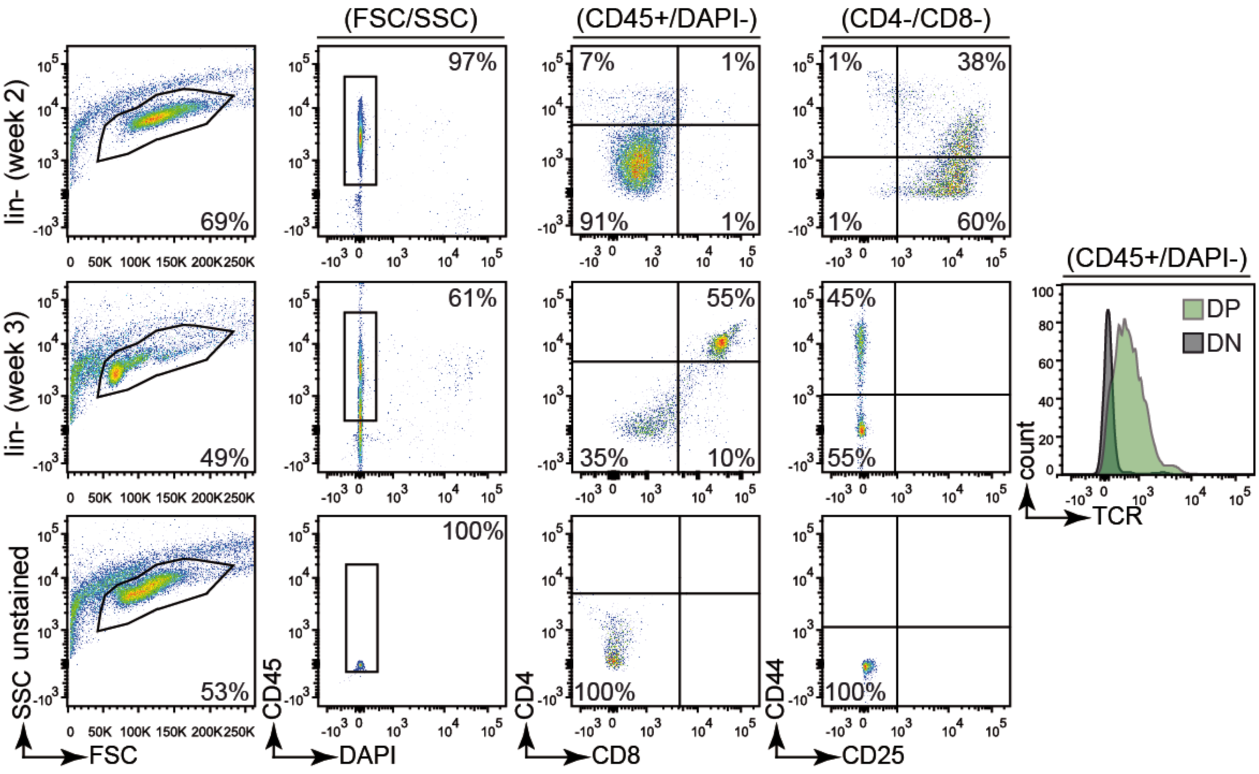

Figure S2. ***In vitro*** **T cell development of lineage-negative cells.** Lin⁻ were co-cultivated on OP9-DL1 monolayers. T cells were harvested and analysed by flow cytometry at weeks 2 and 3. Gating (indicated on top) was applied in the following order: FSC/SSC and CD45+/DAPI- to gate for live hematopoietic cells; CD4-/CD8- or CD4+/CD8+ to gate for double-negative (DN) or double-positive (DP) T cells. Within the DN fraction CD25/CD44 expression was used to assess DN1 to DN4 stages. For lineage-negative cells, TCRβ expression at week 3 was determined in both the DN and DP fraction. Numbers indicate percentage of cells in each quadrant. lin⁻, lineage-negative bone marrow derived hematopoietic stem and progenitor cells.

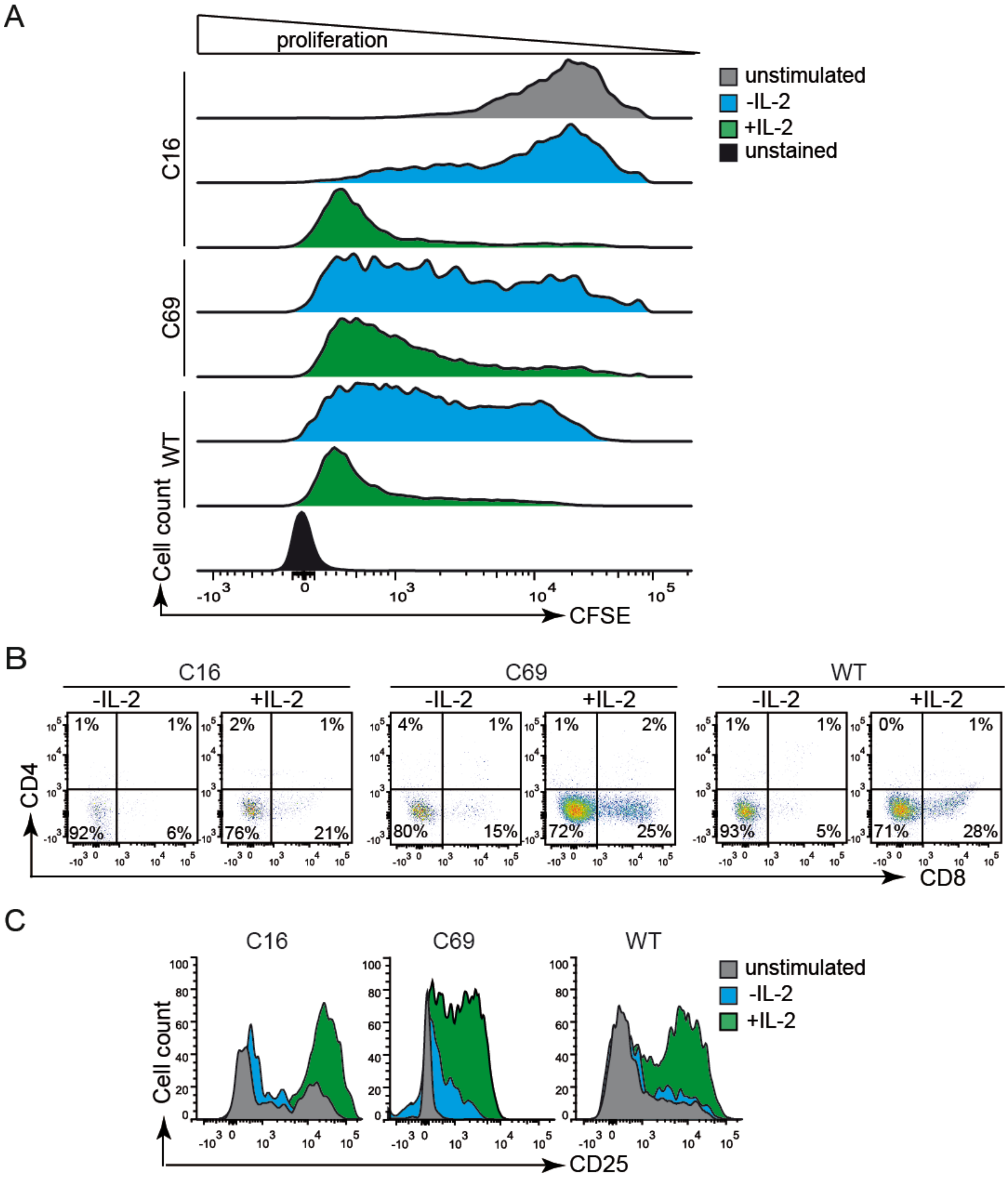

Figure S3. Response of *in vitro* generated T cells to IL-2. Week 4 T lymphocyte population (Figure 3) were co-cultivated for 1 week with OP9-DL1 stroma cells and irradiated antigen-presenting cells (APCs). As co-stimulatory signals, anti-CD3/CD28 beads and IL-2 were provided. (**A**) Proliferation of T cells. T lymphocyte population was labelled with CFSE and activated as described above in the absence (blue) or presence (green) of IL-2. As controls, ‘unstained’ T cells (clone C16) and CFSE-labelled but ‘unstimulated’ T cells (without APCs and beads) were used. After one week, the extend of CFSE staining was evaluated by flow cytometry. CFSE, carboxyfluorescein succinimidyl ester. (**B**) Characterisation of SP CD8+ T cells. T

lymphocyte populations, derived from corrected (C16, C69) and WT ESC clones, were activated as described above in the absence (–IL-2) or presence (+IL-2) of IL-2. CD4/CD8 expression was evaluated after one week by flow cytometry on FSC/SSC-gated cells. (**C**) Activation of SP CD8+ T cells. T lymphocyte populations, derived from corrected (C16, C69) and WT ESC clones, were activated as described above in the absence (blue) or presence (green) of IL-2. CD25 (IL-2 receptor alpha chain) expression was quantified by flow cytometry after one week. Unstimulated, T lymphocyte population cultivated without APCs and anti-CD3/CD28 beads.

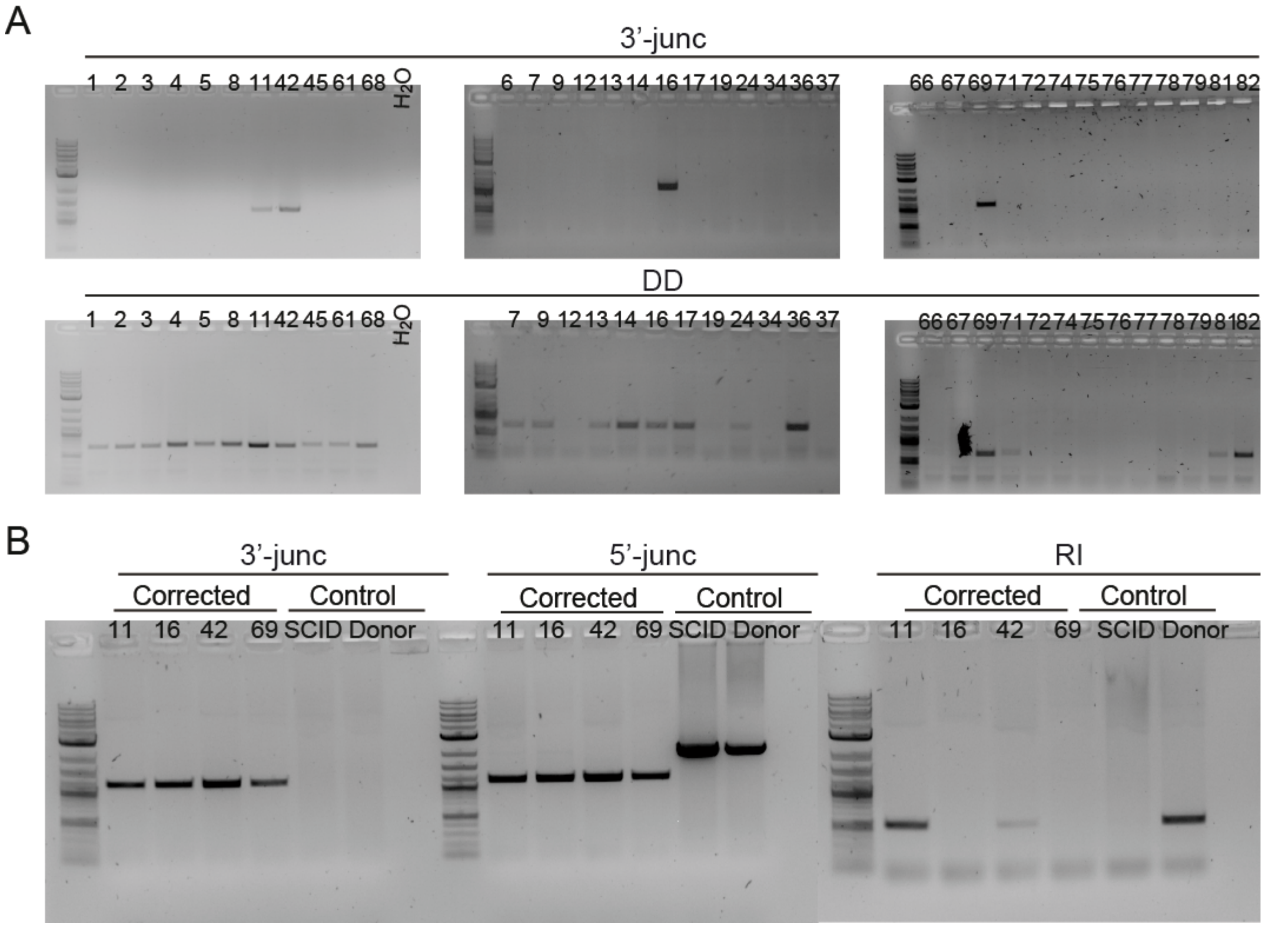

Figure S4. Uncropped images. (**A**) Uncropped images of gels shown in Figure S1. (**B**) Uncropped images of gels shown in Figure 1B.

Table S1. Primers used for genotyping

Purpose	Primer ID	Sequence 5' → 3'	Product size
5'-junction	5'F	TGAACCACTGTTTGGAGCAC	SCID allele: 1.9 kb<br>Gene targeting: 4.1 kb / 1.1 kb
	5'R	AGGTTCTTCAGGGTGGGAAT	
3'-junction	3'F	GGAGGATTGGGAAGAAAATAGCAGGCATGC	1.2 kb
	3'R	ACCCCCACACTCTGTCTGTC	
Donor detection	DDF	AGCTGCAAGAACTCTTCCTCAC	0.7 kb
	DDR	CAGGACGTCGACGCTATTGTCTTCCCAATCCTCCC	
Random integration	RIF	TAATACGACTCACTATAGGG	0.7 kb
	RIR	ACTTCCACAGAGTGGATTGAAGCGGCTCCGAACAC GAAACGTGTAGCGTTTCTG	
Sequencing	E5F	TCAGTGAAGGGAGCAGTGTG	0.5 kb
	E5R	AACAACACGCTAACCCAACC	
